# The Medical Diploma Traffic

**Published:** 1873-04

**Authors:** 


*The Medical Diploma Traffic.*—"It is held by the Supreme Court of Pennsylvania that the repeal of the Eclectic Medical College charter by the Legislature last year is null and void, as being beyond the constitutional power of that body. The case seems to have turned upon the fact that the charter was granted prior to 1857, which says that "the Legislature shall have power to alter, revoke or annul any charter of incorporation *hereafter* conferred," &c. This Eclectic College was one of the establishments charged with selling diplomas for money, instead of issuing them in regular course, based upon study and the competency of the pretended graduate; and the charge, after investigation by a committee of the Legislature, was reported to be well founded. Thereupon the charter was revoked, the Legislature failing to take notice of its date. Judge Agnew says in delivering the opinion of the court, that this particular charter, being older than the constitutional amendment, can be annulled only after trial in a court of competent jurisdiction."

